# The emerging role of lncRNAs in inflammatory bowel disease

**DOI:** 10.1038/s12276-018-0188-9

**Published:** 2018-12-06

**Authors:** Reza Yarani, Aashiq H. Mirza, Simranjeet Kaur, Flemming Pociot

**Affiliations:** 1 Type 1 Diabetes Biology, Department of Clinical Research, Steno Diabetes Center Copenhagen, Copenhagen, Denmark; 2 Department of Pharmacology, Weill Cornell Medicine, Cornell University, New York, NY 10065 USA; 3 Department of Pediatrics, Copenhagen Diabetes Research Center, Herlev University Hospital, Herlev, Denmark; 4 Department of Clinical Medicine, Faculty of Health and Medical Sciences, University of Copenhagen, Copenhagen, Denmark

## Abstract

Dysregulation of long noncoding RNA (lncRNA) expression is linked to the development of various diseases. Recently, an emerging body of evidence has indicated that lncRNAs play important roles in the pathogenesis of inflammatory bowel diseases (IBDs), including Crohn’s disease (CD) and ulcerative Colitis (UC). In IBD, lncRNAs have been shown to be involved in diverse processes, including the regulation of intestinal epithelial cell apoptosis, association with lipid metabolism, and cell–cell interactions, thereby enhancing inflammation and the functional regulation of regulatory T cells. In this review, we aim to summarize the current knowledge regarding the role of lncRNAs in IBD and highlight potential avenues for future investigation. We also collate potentially immune-relevant, IBD-associated lncRNAs identified through a built-by association analysis with respect to their neighboring protein-coding genes within IBD-susceptible loci. We further underscore their importance by highlighting their enrichment for various aspects of immune system regulation, including antigen processing/presentation, immune cell proliferation and differentiation, and chronic inflammatory responses. Finally, we summarize the potential of lncRNAs as diagnostic biomarkers in IBD.

## Inflammatory bowel disease: The role of noncoding RNA

Studying long noncoding RNAs (lncRNAs) may improve diagnosis and treatment of inflammatory bowel disease (IBD). These RNAs are found between genes in DNA regions previously thought to be “junk,” and have recently been shown to be important in development of various diseases. IBD, which includes both Crohn’s disease and ulcerative colitis, damages the digestive tract lining, causing pain and chronic diarrhea. A better understanding of IBD’s complex causes is needed to identify more effective treatments. Flemming Pociot at the Steno Diabetes Center in Gentofte, Denmark, and co-workers reviewed recent research linking lncRNAs and IBD. They discuss how lncRNAs’ roles in immunity and inflammation influence IBD development, describing how particular lncRNAs are related to IBD. Promising avenues for further research are highlighted, including the use of lncRNAs as biomarkers of IBD, which can be difficult to diagnose.

## Introduction

Inflammatory bowel diseases (IBDs) are a group of relapsing chronic inflammatory disorders that affect the gastrointestinal (GI) tract. There are two major forms of IBD: Crohn’s disease (CD) and ulcerative colitis (UC). CD is characterized by patchy transmural inflammatory patterns that are known to affect all layers of the intestinal wall in any part of the GI tract. CD is more common in young individuals; however, it may also occur in older individuals. In general, it is accompanied by several pathophysiological complications, including intestinal fibrosis, abscesses and fistulas, abdominal pain, chronic diarrhea with and without blood, mucus, and fever during highly active disease^1^. UC is a disease of the mucosal lining and is limited to the innermost layers with cryptitis and crypt abscesses. Similar to CD, UC mainly affects a younger population, and it is the most common form of IBD^2,3^. While CD may affect any part of the GI system, UC is limited to the large intestine, with the rectum characterized as the main affected region. UC is less prone to disease-associated complications^3^, and its course is mild in many patients. Both subtypes demonstrate periods of active disease followed by remission, of which each state lasts for variable periods of time.

The etiology of IBD is not well understood; however, comprehensive epidemiologic and genetic studies suggest that IBD is a result of complex interactions between genetics, immune dysregulation, and environmental factors, including lifestyle factors^4^. Genome-wide association studies (GWAS) have identified 163 susceptibility loci in more than 75,000 IBD patients and control individuals of European descent^5,6^. While 110 loci were associated with both phenotypes, 30 loci were unique to CD and 23 loci were unique to UC^5,6^. Genetic predisposition studies have shown an eightfold to tenfold higher risk of IBD in relatives of CD and UC patients^7^. Twin studies have further indicated that when a child has UC or CD, there are 9 and 26 times greater risks, respectively, for the second twin to develop these diseases^8^. However, the current GWAS data seem to account for only 23% and 16% of the heritability in CD and UC, respectively^9^.

The emergence of high-throughput sequencing technologies and advances in transcriptome biology have revolutionized our understanding of gene function and regulation. Remarkably, approximately 98% of the human genome code for RNA molecules were previously not annotated. These RNAs lack the capacity to code for proteins and are therefore termed noncoding RNAs (ncRNAs)^10^. ncRNAs have emerged as important regulators of gene expression at both the transcriptional and post-transcriptional levels. A better understanding of ncRNA mechanisms of gene regulation will increase our knowledge of the physiological and pathological conditions of development, regulation, and function in health and disease processes, which, in turn, opens new avenues for developing novel treatment strategies and nucleic acid diagnostic biomarkers. In this review, we highlight the emerging role of long ncRNAs (lncRNAs) as an important class of ncRNAs in IBD. By reanalyzing our own datasets together with published data, we present the biological processes that are likely impacted by the significantly differentially expressed lncRNAs.

## LncRNAs in IBD

LncRNAs are transcripts that are longer than 200 nucleotides and can be processed similar to protein-coding mRNAs by RNA polymerase II^11^. To date, there is no clear or final classification for lncRNAs. Based on the proximity to protein-coding mRNAs, they can be assigned to one or more categories, including sense and antisense lncRNAs, bidirectional lncRNAs, intronic lncRNAs, and long intergenic ncRNAs^12^. Our knowledge regarding the number of lncRNA transcripts is also not consistent. While studies have reported approximately 20,000 lncRNAs in the human genome^13^, according to the NONCODE database’s latest version 5 (2018), there are 172,216 lncRNA transcripts and 96,308 lncRNA genes for humans. These numbers of transcripts and genes are substantially higher than the 28,468 lncRNA loci transcripts and 15,779 lncRNA genes according to GENCODE version 28 (November 2017 freeze, GRCh38) – Ensembl 92. While these statistical disagreements could simply be the result of the difference in updates or disagreement regarding gene/transcript categorization, it also reflects the lack of knowledge regarding this class of macromolecules. The functions and mechanisms of action of most lncRNAs are not fully understood; however, based on the current findings, they exhibit diverse functional roles, including the regulation of protein-coding genes by chromatin remodeling, modulation of gene expression, regulation of protein activity, localization, and stability^13–16^. The role of lncRNAs in immune function regulation and the progression of autoimmune diseases, including type 1 diabetes^17^, rheumatoid arthritis^18^, osteoarthritis^19^, psoriasis^20^, and asthma^21^, is gaining increasingly more attention. Although the pathological/diagnostic roles of microRNAs (miRNAs) as another class of ncRNAs (small RNAs with a size of 20–25 nucleotides) have been the focus of many IBD studies^22–24^, the potential roles of lncRNAs remain largely unknown. However, several recent studies have investigated the association of dysregulated lncRNAs with both CD and UC (summarized in Table 1) using colonic biopsies or blood samples (or both) and have further highlighted their roles in disease-related processes, including the regulation of intestinal epithelial cells and inflammation.

**Table 1:** Known lncRNAs dysregulated in IBD

LncRNA	Disease/population	Sample source	Assay method	Fold change^1^/*P* value	Comments	Ref.
DQ786243^2^	aCD/11 iCD/8 CO/9	PBMCs (blood)	qRT-PCR	aCD vs. iCD: *P* = 0.012aCD vs. CO: *P* = 0.002	• Regulates the function of Treg through CREB and Foxp3—relates to the severity of CD	^25^
CDKN2B-AS1 (ANRIL)	CD/13 UC/20 CO/12	Colonic tissue	Microarray qRT-PCR	UC: −8.31 *P* < 0.05CD: −2.97 *P* < 0.05	• Haberman et al. (2018): Transcriptional regulatory role in intestinal epithelia (FC: −7.4) • Pothoulakis et al. (2017): Associate with lipid metabolism process and with cell–cell interactions (FC: −12 in UC and −4.9 CD)	^33, 36^
IFNG-AS1	aUC/8 iUC/4 CO/7	Colonic tissue	Microarray qRT-PCR	5.27*P* = 7.07E−06	• An independent validation cohort of 16 control and 15 UC patients was used • Enhancer of inflammation by regulating IFNG inflammatory responses in CD4 T cells	^38^
H19	UC/12 CO/PANT	Colonic tissue	qRT-PCR	4.45*P* < 0.05	• H19 upregulation causes miR-675-5p increased expression and vitamin D receptor and tight junction proteins decreased expression—destruction of intestinal epithelial barrier function	^47^
BC012900^2^	aUC/16 iUC/15 CO/15	Sigmoid colonic tissue	Microarray qRT-PCR	*P* < 0.001	• Regulates intestinal epithelial cell apoptosis • Out of six top lncRNAs in this study, four were not found on the current built of the human reference genome (GRCh38)	^13^
BC062296 (B3GALT5-AS1)	aUC/16 iUC/15 CO/15	Sigmoid colonic tissue	Microarray qRT-PCR	*P* < 0.05	• Downregulated in the UC • No function is suggested for this lncRNA • Out of six top lncRNAs in this study, four were not found on the current built of the human reference genome (GRCh38)	^13^
GUSBP2	CD/12 CO/12	Plasma (blood)	Microarray qRT-PCR	626.49*P* < 0.05	• Among the lncRNAs 15 were antisense, 81 were enhancer and 161 were lincRNAs	^50^
AF113016 (MALAT1)	CD/12 CO/12	Plasma (blood)	Microarray qRT-PCR	−481.03*P* < 0.05	• Among the lncRNAs 15 were antisense, 81 were enhancer and 161 were lincRNAs	^50^
CCAT1 (CASC19)	UC/8 iUC/4 CO/7	NCM460 cells Colonic tissue	Microarray	NCM460-NTR1: 2.3 *P* = 0.001UC: 11.9*P* = 0.0004	• Human colonic epithelial cells (NCM460) where NTR1 is overexpressed was stimulated by neurotensin • Cohort of IBD patients and normal controls were from Padua et al. (2016) • Cross-compression of the lncRNA from in vitro and in vivo studies revealed CCAT1 and UCA1 were the two with highest significant overexpression	^57^
UCA1	aUC/8 iUC/4 CO/7	NCM460 cells Colonic tissue	Microarray	NCM460-NTR1: 2.9 *P* = 0.002UC: 2.3*P* = 0.002		^57^
HNF4A-AS1	CD/111 CO/30	Ileal biopsy	RNA-seq qRT-PCR	–6.1*P* < 0.05	• An independent validation cohort of 8 control and 28 CD patients was used • Association with an epithelial metabolic signature • Unique to ileal cohort • 314 downregulated coexpressed genes	^33^
RP11-44K6.2	CD/111 CO/30	Ileal biopsy	RNA-seq qRT-PCR	5.3*P* < 0.05	• An independent validation cohort of 8 control and 28 CD patients was used • Also in adult colon • Mirza et al. (2015): in CD FC: 3.83	^33^
LINC01272 (SMIM25)	CD/84 UC/84 CO/84	Plasma (blood) Colonic tissue	qRT-PCR	*P* < 0.001	• LINC01272 expression in CD tissue and plasma (*R*^2^ = 0.7133; *P* < 0.0001) • LINC01272 expression in UC tissue and plasma (*R*^2^ = 0.5326, *P* < 0.0001) • Haberman study: Also in adult colon (FC: 9.4), association with myeloid proinflammatory signature and immune activation functions • 187 upregulated coexpressed genes • Mirza et al. (2015): in CD FC: 3.35	^70^
KIF9-AS1	CD/84 UC/84 CO/84	Plasma (blood) Colonic tissue	qRT-PCR	*P* < 0.001	• KIF9-AS1 expression in CD tissue and plasma (*R*^2^ = 0.3788, *P* = 0.0002) • KIF9-AS1 expression in UC tissue and plasma (*R*^2^ = 0.3466, *P* = 0.0012)	^70^
DIO3OS	CD/84 UC/84 CO/84	Plasma (blood) Colonic tissue	qRT-PCR	*P* < 0.001	• DIO3OS expression in CD tissue and plasma (*R*^2^ = 0.2524, *P* = 0.0141) • DIO3OS expression in UC tissue and plasma (*R*^2^ = 0.2707, *P* = 0.0083) • Chen, Liu et al. (2016) study: Upregulated, FC: not mentioned • Other studies: downregulated, for example, Mirza et al. (2015): in CD FC: −3.01	^70^
GAS5	UC/16 CD/3	PBMC (blood) HeLa and LoVo cells	qRT-PCR	Over all: *P* = 0.033	• Patients treated with prednisone 1–2 mg/kg per day for 30 days • Resistance to glucocorticoid (GC) treatment • Candidate marker of GC resistance and consequently progression of the disease	^72^

*UC* ulcerative colitis, *CD* Crohn’s disease, *CO* control, *a* active, *i* inactive, *PBMC* peripheral blood mononuclear cell, *PANT* paired adjacent normal tissues, *NA* not available

Note: Some lncRNAs have either dual annotation or their annotation is changed in the current built of the human reference genome (GRCh38)

^a^Fold change values mainly relative to normal controls, otherwise stated

^b^Changed annotation based on the current built of the human reference genome (GRCh38), now protein-coding genes

## DQ786243

One of the first reports elucidating the role of lncRNAs in IBD was the study by Qiao et al^25^. They showed that in both disease active and inactive CD patients, DQ786243 lncRNA is upregulated compared to control subjects. However, quantitative reverse transcription-PCR (RT-PCR) indicated a significant overexpression of DQ786243 in active CD patients compared with inactive CD patients and healthy controls^25^. Subsequent overexpression of DQ786243 in Jurkat cells has further shown that DQ786243 can regulate the function of regulatory T lymphocytes (Treg) through changes in the expression of Treg-related cAMP response element-binding protein (CREB) and fork head box P3 (Foxp3). It is established that defective Tregs play an important role in CD pathogenesis^25^. In addition, it has been suggested that lncRNA DQ786243 regulates many biological processes, including the cell cycle, apoptosis, metastasis, proliferation, and invasion, and G2/M cell cycle arrest in colorectal cancer. More recently, the contribution of DQ786243 to the proliferation and metastasis of colorectal cancer as an oncogene that promotes tumor progression both in vitro and in vivo has been elucidated. In vitro knockdown of DQ786243 inhibits cell proliferation, invasion, and migration^26^. Therefore, authors suggest that dysregulation of DQ786243 is likely to play an important role in the pathogenesis of both CD and colorectal cancers, whereas its role in UC remains elusive. However, DQ786243 based on the current build of the human reference genome (GRCh38) has a changed annotation and is categorized as a protein-coding gene.

## CDKN2B-AS1 (ANRIL)

CDKN2B-AS1, which is also referred to as ANRIL, is a 3.8K nucleotide-long antisense lncRNA located in the INK4 locus that has been identified within the p15/CDKN2B-p16/CDKN2A-p14/ARF gene cluster and is associated with multiple human diseases^27^. It is encoded on the chromosome 9p2.3 region and has emerged as a regulatory molecule in many different human diseases^28–31^. In contrast to cancers, where CDKN2B-AS1 showed significant upregulation, in the IBD pathology context, it shows a significant downregulation^13,32,33^. In our previous study, we found that out of 17 annotated ANRIL isoforms, 8 major isoforms were downregulated in inflamed UC colon pinch biopsies (fold change (FC) <−7.9, −8.31, *P* value <0.05) and inflamed CD (FC <−2.7, −2.97, *P* value <0.05) compared with noninflamed and control tissues, respectively^32^. One of the eight isoforms was circular variant (cANRIL), which was universally downregulated in both inflamed CD and UC. It has been shown that circular RNAs act as miRNA sponges and are involved in stabilizing sense transcripts^34^. However, their biological functions require further investigation to be fully understood. Most recently, Haberman et al.^33^ identified CDKN2B-AS1 as one of the top 15 downregulated lncRNAs in the ileum of treatment-naive early-onset CD patients. They found 411 downregulated genes to be coexpressed with this lncRNA in the cohort, which was the highest coexpression number compared with the other discovered lncRNAs. They further identified a significant decrease in the expression of CDKN2B-AS1 after interleukin-1β (IL-1β) stimulation of Caco-2 cells. CDKN2B-AS1 is enriched in the nuclear fraction, which suggests a transcriptional regulatory role of this lncRNA in intestinal epithelial cells. Moreover, in a gene expression profiling study that included eight UC, seven CD, and seven control colonic samples, Rankin et al.^35^ found that CDKN2B-AS1 lncRNA was significantly downregulated by 4.9-fold (*P* value: 0.041) in CD patient samples and 12-fold (*P* value: 0.0009) in UC samples compared to control samples. These findings were validated in independent colon samples of 16 controls and 15 UC patients. Investigation of the CDKN2B-AS1 expression in several colon epithelial cell lines, including NCM460, HCT116, and DLD-1, showed a positive correlation with N-myc as an important regulatory transcription factor, which was also confirmed in IBD patient samples (*R*^2^: 0.691; *P* value: 0.004304). Furthermore, while tumor necrosis factor-α (TNF-α) and IL-1β treatment in the NCM460 cell line did not affect the CDKN2B-AS1 expression, tumor growth factor-β (TGF-β) treatment resulted in a twofold (*P* value: 0.0289) downregulation of this lncRNA. This observation was fully compatible with the inverse correlation of TGF-β1 expression in human samples (*R*^2^: 0.573; *P* value: 0.0255). Through gene pathway analysis, it has been further shown that CDKN2B-AS1 lncRNA is associated with lipid metabolism and cell–cell interactions^36^. However, to elucidate the effect of dysregulated CDKN2B-AS1 on colonic epithelial cell functions in IBD, for example, apoptosis proliferation and barrier formation, further studies are required.

Furthermore, Zhou et al.^27^ showed CDKN2B-AS1 regulates inflammatory responses as a novel component of the nuclear factor-κB (NF-κB) pathway. Following CDKN2B-AS1 knockdown in umbilical vein endothelial cells, the expression of CDKN2A/B as a neighboring gene was assessed. It is known that CDKN2A/B encode for the cell cycle regulators p15^INK4B^ and p16^INK4A^, which is epigenetically regulated by CDKN2B-AS1^37^. They found that not only is cell growth arrested but the RNA levels of p15^INK4B^ and p16^INK4A^ are also upregulated where CDKN2B-AS1 is knocked down, which shows the repressive effect of this lncRNA, as well as its regulatory effect on cell proliferation. These findings were also confirmed by chromatin immunoprecipitation assay. They further investigated the role of CDKN2B-AS1 under pathological conditions by measuring its expression following proinflammatory cytokine (TNF-α) and growth factor (VEGF165 and PDGF-BB) stimulation. While the CDKN2B-AS1 expression showed a remarkable increase (~5-fold) following TNF-α stimulation (in contrast to ref. ^35^), no detectable change was identified following growth factor treatment. Further RNA-sequencing indicated that CDKN2B-AS1 silencing dysregulated the IL-6 and IL-8 expression under TNF-α treatment. They further showed Yin Yang 1, which is a CDKN2B-AS1-binding transcriptional factor, is required for IL-6/8 expression under TNF-α treatment. The differences in the dysregulation of CDKN2B-AS1 (and specifically cANRIL) are highly fascinating and may be an interesting case for further investigations to better understand the role of this lncRNA in IBD pathogenesis.

## IFNG-AS1

In a recent study, a subset of 31 differentially expressed lncRNAs in UC patients (out of 767 evaluated lncRNAs) was found to be within 250 kb from the IBD susceptibility loci. IFNG-AS1 was significantly differentially expressed between UC and control colonic samples. A UC phenotype associated single-nucleotide polymorphism (SNP), rs7134599, located adjacent to the inflammatory cytokine interferon-γ (IFN-γ)^38^, seems to influence the IFNG-AS1 expression as expression quantitative trait loci. This finding confirms our previous study that showed IFNG-AS1 was upregulated in inflamed UC vs. control subjects (FC = 1.54) and noninflamed UC (FC = 1.52)^32^. Both studies also showed IFNG-AS1 located adjacent to the key inflammatory cytokine and ILs, such as IFN-γ, IL-22, and IL-26. IFNG-AS1, which was originally named NeST or Theiler’s murine encephalomyelitis virus persistence candidate gene 1, is encoded on the opposite DNA strand to the *IFNG* coding strand^39^ and is one of the most widely studied lncRNAs. Through pathway analysis, an increase in the expression of several upstream inflammatory response regulators, including IFN-γ, IL-1, IL-6, and TNF-α, in UC patients with high IFNG-AS1 levels has been elucidated. It has also been shown that IFNG-AS1 acts as an enhancer of inflammation by acting as a novel regulator of IFN-γ inflammatory responses in CD_4_ T (Th1) cells in UC patients with a high expression of IFNG-AS1^38^. This finding clearly suggests the importance of the lncRNA IFNG-AS1 on the regulation of UC disease-related inflammatory responses and suggests that lncRNA can be used to differentiate active UC patients from remission phase patients and the healthy population. In addition, IFNG-AS1 has been implicated in other autoimmune diseases, including Hashimoto’s thyroiditis (HT) and asthma^40–42^. In HT, the IFNG-AS1 expression was correlated with IFN-γ expression, T cell response, and autoantibody titers^40^. The role of IFNG-AS1 in regulating the pathophysiology of several autoimmune-related diseases, through similar or common processes (e.g., regulating the expression of IFN-γ^43,44^), clearly suggests the importance of lncRNAs for further analysis.

## H19

H19 has been extensively investigated in cancer biology^45,46^, while its role in IBD and other inflammatory or autoimmune diseases has been less studied. The only available study in IBD was performed by Chen et al. ^47^, in which the role of H19 in UC development was investigated, and the potential correlation of H19 with intestinal epithelial barrier function was assessed. They showed that the expression of H19 in colonic biopsies is negatively correlated with the expression of vitamin D receptor (VDR) in UC. VDR signaling plays an important role in inflammation regulation^48^. One important mechanism in volcanic intestinal inflammatory responses in UC development and propagation has been demonstrated to be the decrease in VDR expression^47^. Interestingly, overexpression of H19 resulted in significantly decreased expression of the VDR (mean: 0.37; *P* < 0.05) and had a destructive effect on the intestinal epithelial barrier function by increasing permeability (in vitro model of intestinal epithelium using Caco-2 monolayer) and decreasing the expression of both main tight junction proteins (ZO-1 and occludin)^47^. The study clearly supports a role of H19 lncRNA in the development of UC. Zou et al.^49^ further showed that the effect of lncRNA H19 on intestinal epithelial barrier function is accomplished by serving as a precursor for miRNA-675. The overexpression of H19 increased the miRNA-675 abundance (mean: 1.897; *P* < 0.01), which, in turn, downregulated the adherent junction proteins E-cadherin and ZO-1 by repressing and destabilizing their translation. They further showed that targeted deletion of RNA-binding protein HuR enhanced miRNA-675 production in the mucosa, while increasing the level of this protein overexpressing H19 prevented the stimulation of miRNA-675. Therefore, they speculate H19 interacts with HuR and regulates its function via the H19-encoded miRNA-675.

## BC012900 and BC062296

H19 is not the only lncRNA that regulates epithelial cell function. Intestinal epithelial cell apoptosis has also been shown to be regulated by BC012900, which is also a UC-associated lncRNA^13^. Among 329 upregulated lncRNAs identified in active UC compared with healthy controls, the three most significantly upregulated lncRNAs were BC012900, AK001903, and AK023330. Moreover, out of 126 downregulated lncRNAs, the three most significantly downregulated lncRNAs were the BC029135, CDKN2B-AS1, and BC062296 transcripts. Among these lncRNAs, the authors decided to perform further studies on BC012900 expression. Thus, using quantitative PCR (qPCR) and in situ hybridization, Wu et al.^13^ showed that the lncRNA BC012900 is significantly differentially expressed in active UC compared to control subjects and is localized in the nucleus. Stable overexpression of BC012900 stimulated by inflammatory cytokines in epithelial cell lines (HCT116 and HT29) resulted in a significant inhibition of cell proliferation and a significant increase in apoptosis that were positively correlated with the expression level. Therefore, BC012900 may represent an interesting target for future therapeutics or diagnostic biomarker development in IBD. Among the investigated lncRNAs, BC012900 was uniquely expressed in active UC compared with other conditions. They also examined the downregulated BC062296 localization in an epithelial cell line and showed that it is highly abundant in the cytoplasm, which suggests its different regulatory activity compared to BC012900 that was found in the nucleus. However, in situ hybridization of BC062296 showed that in contrast to BC012900, it is localized in both the cytoplasm and nucleus. Despite these findings and observations, five out of six top up- and downregulated lncRNAs in this study were not found on the current build of the human reference genome (GRCh38) or had changed annotations as protein-coding genes, and BC062296 is the only available lncRNA.

## GUSBP2, AF113016, and DIO3OS

In a recent study by Chen et al.^50^, microarray screening of CD patient plasma indicated more than 1200 and 1000 upregulated and more than 700 and 900 downregulated lncRNAs and mRNAs, respectively. qRT-PCR evaluation confirmed the findings of the microarray screening, and the highest up- and downregulated fold changes of lncRNAs were shown to be for GUSBP2 and AF113016, respectively. The study was the first investigation to evaluate plasma lncRNAs as biomarkers for the diagnosis of CD. Primarily, 1988 and 2993 dysregulated lncRNAs and mRNAs between the CD and control groups were identified in this study, which is substantially higher than the 450 lncRNAs and 1100 mRNAs from our own study in 2015^32^. Moreover, in contrast to our previous study, which showed that DIO3OS is downregulated^32^, in Chen et al.’s study, this lncRNA was upregulated. Another disagreement between the two studies was the lack of overlap of dysregulated lncRNAs between our top 10 dysregulated results and their results. One potential explanation for the differences in the studies could be the different sources, as we used colonic samples and they used plasma.

## CCAT1 and UCA1

A 13 amino acid length neuropeptide, neurotensin (NT), together with its high affinity G protein-coupled receptor 1 (NTR1), are believed to promote the development of UC and are expressed in the colon where they are upregulated in animal colitis models and UC patients^51–54^. NT/NTR1 signaling is involved in various colonic responses, including proinflammatory responses, and regulates several miRNAs associated with intestinal inflammation^55,56^. Law et al.^57^ investigated the effect of NT stimulation on the lncRNA profile of human colonic epithelial cells (NCM460) with NTR1 overexpression. In this study, microarray data initially showed 150 and 155 down- and upregulated lncRNAs, respectively. A comparison of the lncRNAs identified in this study with the lncRNAs found in the study by Padua et al.^38^ indicated that 13 lncRNAs of 305 are commonly dysregulated in these two studies. The CCAT1 and UCA1 lncRNAs were found to be significantly overexpressed compared to those identified from human UC patients and control subjects. Both UCA1 and CCAT1 lncRNAs play an important role in the pathogenesis of many diseases, including various types of cancers and inflammation-related disorders^25,58–63^. UCA1, also referred to as CUDR, was first detected in gastric juice. Its abnormal expression is always accompanied by various types of human cancers^64,65^. Studies have shown that these two lncRNAs act as sponges for let-7 and miR-204^66,67^, which are miRNAs downregulated by NT/NTR1 signaling in human colonic NCM460-NTR1 cells^68^. Furthermore, CCAT1 modulated the HOXA1 expression and promoted disease progression through sponging miR-181a-5p in multiple myeloma^69^. Since limited data exist to evaluate the role of NTR1 in human colonic epithelial cells and its potential in UC pathophysiology (e.g., through the lncRNA–miRNA axes), further studies are required^57^.

## HNF4A-AS1, LINC01272, and RP11-44K6.2

In a new study, Haberman et al.^33^ characterized the lncRNA landscape in the ileum of treatment-naive, that is, not influenced by treatment, early-onset pediatric CD patients. In this study, which was performed in pediatric CD patients, 111 ileal biopsies were profiled. They showed widespread dysregulation of more than 450 lncRNAs in patients compared with healthy control subjects. For prioritization of differentially expressed lncRNAs, fold change differences between CD and healthy control subjects were initially considered. From the top 15 up- and downregulated lncRNAs, CDKN2B-AS1, HNF4A-AS1, LINC01272, and RP11-44K6.2 were selected. These lncRNAs were selected because they showed up in both adult (from the same group’s previous study) and pediatric cohorts, had the highest number of coexpressed genes, and/or were unique. CDKN2B-AS1 was shown to be one of the lncRNAs with the highest coexpressed genes. CDKN2B-AS1 and HNF4A-AS1 showed a significant downregulation in the Ileum of the treatment-naive cohort, whereas the downregulation of HNF4A-AS1 lncRNA was unique to this study and was not shown elsewhere. In contrast to the previous two lncRNAs, LINC01272 and RP11-44K6.2 showed a significant upregulation in this cohort, as well as in the adult colon. In addition, LINC01272 demonstrated the highest number of upregulated coexpressed genes of the top 15 upregulated lncRNAs. A coexpression analysis was performed with a well described, large set of genes using Pearson's correlation analyses (*r* > 0.75) as a read out for the potential functionality of the top 15 lncRNAs. Moreover, they showed the tissue-specific expression of these lncRNAs in biopsy samples using in situ hybridization and suggest that lncRNAs demonstrate more tissue-specific expression patterns compared with protein-coding genes. While HNF4A-AS1 showed epithelial-specific expression, LINC01272 showed specific myeloid expression. To identify the biological processes, networks/pathways, molecular functions, and phenotypes, functional annotation enrichment analyses using groups of related genes were performed for the selected lncRNAs. It has been shown that LINC01272 is associated with immune activation functions, whereas HNF4A-AS1 is associated with metabolic functions. HNF4A-AS1 and LINC01272 expression showed significant correlations with more severe mucosal injury when correlation is tested with clinical disease activity indices and the severity of mucosal injury. These findings were confirmed using RT-qPCR in the validation cohort. Finally, the authors suggest that the significant correlations between these lncRNA expressions and mucosal tissue injury highlight the need to further investigate the role of these two lncRNAs in CD pathogenesis^33^.

## KIF9‑AS1, LINC01272, and DIO3OS

In a very recent study, three lncRNAs, KIF9‑AS1, LINC01272, and DIO3OS, were selected for evaluation as potential diagnostic biomarkers for IBD^70^. LINC01272 and DIO3OS were also identified by previous studies^32,33,50^. The expression levels of these lncRNAs in colonic tissue and plasma samples of 84 UC, 84 CD, and 84 healthy individuals were investigated. The results showed significantly higher expression levels of KIF9‑AS1 and LINC01272 in UC/CD patients than in healthy individuals (*P* < 0.001) in both colonic and plasma samples. The specificity and sensitivity of these two lncRNAs were evaluated by a receiver-operating characteristic (ROC) curve analysis in both UC and CD and compared with healthy controls. The area under the ROC curve for KIF9‑AS1 expression in CD and UC vs. healthy controls was 0.811 (*P* < 0.0001) and 0.872 (*P* < 0.0001), respectively, and 0.887 (*P* < 0.0001) and 0.777 (*P* < 0.0001), respectively, for LINC01272. In contrast, the expression level of DIO3OS was significantly lower in CD/UC patients than in healthy controls (*P* < 0.001) in both colonic and plasma samples, which confirms our previous findings^32^. The area under the ROC curve between the DIO3OS expression in CD and UC and healthy individuals was also reported as 0.794 (*P* < 0.0001) and 0.653 (*P* = 0.001), respectively. Finally, the correlations between colonic biopsy and plasma sample expression levels of these three lncRNAs were investigated by Pearson's correlation coefficient. The results showed a positive correlation between the detected expression in tissue and blood for all lncRNAs in both CD and UC patients, and the ROC curve assessment also confirmed the diagnostic value of these lncRNAs. Therefore, the authors suggest that these three lncRNAs are robust candidates for being considered as IBD biomarkers.

## GAS5

LncRNAs can also cause resistance to treatment and increase the severity of IBD. Growth arrest-specific transcript 5 (GAS5) is a lncRNA encoded at 1q25 and is important in both nontransformed lymphocytes and T cell lines’ cell cycle, normal growth arrest, and apoptosis^71^. In a recent study using both cell lines and IBD patient samples, an increased level of lncRNA GAS5 was identified after treatment with glucocorticoids (GCs). A significant increase in the level of GAS5 was shown to be the rate-limiting factor in the remission of at least 20% of IBD patients showing resistance to GC treatment^72^. GCs are one of the first line medications in the treatment of both main types of IBD. However, considerable interindividual variability in the responses of IBD patients to GC treatment has been documented^73^. GAS5 is an lncRNA encoded by the *GAS5* gene, which acts as the riborepressor of the GC receptor^74^. Upregulated GAS5 binds to the DNA-binding domain of the GC receptor, decoying the receptor away from its GC response element and therefore results in GC resistance by inhibiting transcriptional responses to GCs^74^. GAS5 is sensitive to upframeshift 1, an RNA helicase for nonsense-mediated mRNA decay (NMD), which suggests that some lncRNAs can be degraded through NMD^75^. It has been shown that GAS5 is differentially expressed in cell lines before and after methylprednisolone treatment and is positively correlated with drug resistance. A 4-week treatment of 19 pediatric IBD patients who were categorized as steroid-resistant (SR), steroid-sensitive (SS), or steroid-dependent (SD) showed that SR and SD patients had higher levels of GAS5 expression than the SS group^72^. As a result of these findings, the authors suggest that GAS5 expression can be considered a candidate marker of GC resistance. Nevertheless, additional studies are required to elucidate the exact role of this lncRNA in disease progression.

## IBD-loci-associated lncRNAs

In a previous study of genome-wide transcriptome profiling of lncRNAs and protein-coding genes in 96 colon pinch biopsies, we identified widespread dysregulation of lncRNAs and protein-coding genes in inflamed and noninflamed colon tissues in CD and UC compared to healthy control subjects^32^. In total, 438 differentially expressed lncRNAs in inflamed CD and 745 in inflamed UC were identified compared with that in the control group. In contrast, only 12 and 19 differentially expressed lncRNAs were identified in cases of the noninflamed CD and UC tissues, respectively, compared with the control group. This study allows us to stratify CD from UC and the healthy control population by highlighting the differential expression of lncRNAs and the protein-coding genes in inflamed CD and UC.

Moreover, an extensive landscape of functional mutations in the noncoding genome has been identified from GWAS with profound effects on lncRNA expression^76^. SNPs can lead to complete or partial changes in lncRNA functions. For example, they can result in the loss of miRNA-mediated lncRNA degradation and consequently increased lncRNA expression^77^. Interestingly, in our previous study, significant enrichment for 154 protein-coding genes and 96 differentially expressed lncRNAs was observed within the IBD GWAS loci^32^. The majority of the GWAS signals map to the noncoding intronic and intergenic regions^5^. The differentially expressed lncRNAs colocalized with 44 IBD-risk variants and were also found to be enriched within the IBD loci (*P* < 0.0001). These IBD GWAS loci-associated lncRNAs included H19, SMIM25, and IFNG-AS1, which were also identified as dysregulated in IBD by other studies^38,47,70^.

## Guilt-by-association analysis

Based on our previous study and a literature search, we identified differentially expressed lncRNAs in IBD. Forty-seven lncRNAs associated with IBD were identified (Table 2). From our previous study, 46 lncRNAs (96 transcripts) that were differentially expressed in CD and UC and associated with IBD loci were selected (Gene Expression Omnibus database: GSE67106)^32^. Of these lncRNAs, 10 lncRNAs were deprecated or changed annotation in the current genome build (GRCh38) and were therefore removed. In addition, the three lncRNAs most differentially expressed and not mapping on IBD loci from our top 10 lists were added. Based on the literature search, 19 significantly differentially expressed lncRNAs were selected, and five of these lncRNAs were not found or changed annotations (e.g., DQ786243 and BC012900). Therefore, 14 lncRNAs were selected, of which six were common to our study. Three (KIF9-AS1, ANRIL (CDKN2B-AS1), and DIO3OS) of the six common lncRNAs were in our top 10 dysregulated lncRNAs. In our study, in both CD and UC, KIF9-AS1 was in the top 10 upregulated and CDKN2B-AS1 was in the top 10 downregulated lncRNAs, whereas DIO3OS showed significant downregulation only in the CD patients. Neither of these three lncRNAs are associated with IBD susceptible loci.

**Table 2:** List of IBD-associated lncRNAs alongside their 100 kb up- and downstream neighboring protein-coding genes

ID	Name	PMID	Protein-coding gene (100 kb)
Upregulated in CD and UC			
ENSG00000234741	***GAS5***	28722800	*CENPL*, *DARS2*, *KLHL20*, *RC3H1*, *SERPINC1*, *ZBTB37*
ENSG00000227398	***KIF9-AS1***	29207070, 25991924	*KIF9*, *KLHL18*, *SETD2*
ENSG00000224397	*SMIM25*	29207070, 25991924	*CEBPB*
ENSG00000204044	*SLC12A5-AS1*	25991924	*CD40*, *MMP9*, *NCOA5*, *PCIF1*, *SLC12A5*, *ZNF335*
ENSG00000204261	*PSMB8-AS1*	25991924	*HLA-DMB*, *HLA-DOB*, *HLA-DQA2*, *HLA-DQB2*, *PSMB8*, *PSMB9*, *TAP1*, *TAP2*, *XXbac-BPG181M17.5*, *XXbac-BPG246D15.9*
ENSG00000206337	*HCP5*	25991924	*ATP6V1G2*, *ATP6V1G2-DDX39B*, *DDX39B*, *HLA-B*, *LTA*, *MCCD1*, *MICA*, *MICB*, *NFKBIL1*, *TNF*
ENSG00000226032	*AL035530.1*	25991924	*RSPH3*, *TAGAP*
ENSG00000249086	*AC051649.1*	25991924	*LSP1*, *MRPL23*, *PRR33*, *SYT8*, *TNNI2*, *TNNT3*
ENSG00000235641	*LINC00484*	25991924	*AUH*
ENSG00000261040	*WFDC21P*	25991924	*CA4*, *HEATR6*, *USP32*
ENSG00000268734	*AC245128.3*	25991924	*FCAR*, *KIR2DL4*, *KIR3DL1*, *KIR3DL2*, *NCR1*, *NLRP2*, *NLRP7*
ENSG00000269489	*AL589765.6*	25991924	*C2CD4D*, *CELF3*, *LINGO4*, *MRPL9*, *OAZ3*, *RIIAD1*, *RORC*, *SNX27*, *TDRKH,* *THEM4*, *THEM5*
ENSG00000232807	*AL137186.2*	25991924	*ANKRD16*, *FBXO18*, *GDI2*, *IL15RA*, *IL2RA*
Downregulated in CD and UC			
ENSG00000258498	***DIO3OS***	29207070, 25991924	*DIO3*
ENSG00000240498	***CDKN2B-AS1***	25991924	*CDKN2A*, *CDKN2B*, *MTAP*, *RP11-145E5.5*
ENSG00000233006	*MIR3936HG*	25991924	*C5orf56*, *P4HA2*, *PDLIM4*, *SLC22A4*, *SLC22A5*
ENSG00000259347	*AC087482.1*	25991924	*SMAD3*
ENSG00000259970	*AC099668.1*	25991924	*AMIGO3*, *APEH*, *BSN*, *GMPPB*, *IP6K1*, *MST1*, *RNF123*
ENSG00000264269	*AC016866.1*	25991924	*DYM*, *SMAD7*
Upregulated in UC			
ENSG00000255733	*IFNG-AS1*	27492330, 25991924	*IFNG*, *IL22*, *IL26*, *MDM1*
ENSG00000130600	*H19*	27661667, 25991924	*MRPL23*, *TNNT3*
ENSG00000212978	*AC016747.1*	25991924	*AHSA2*, *C2orf74*, *KIAA1841*, *PEX13*, *USP34*
ENSG00000224220	*AC104699.1*	25991924	*DNMT3A*, *DTNB*
ENSG00000273782	***UCA1***	–	*CYP4F11*, *CYP4F2*, *OR10H1*, *OR10H3*, *OR10H5*
ENSG00000254166	***CASC19***	–	
ENSG00000225582	*AC011193.1*	25991924	
ENSG00000229425	*AJ009632.2*	25991924	
ENSG00000232698	*AP001058.1*	25991924	*AIRE*, *C21orf33*, *DNMT3L*, *ICOSLG*, *PFKL*, *PWP2*, *TRAPPC10*
ENSG00000237499	*AL357060.2*	25991924	*TNFAIP3*
ENSG00000264968	*AC090844.2*	25991924	*CSF3*, *GSDMA*, *GSDMB*, *IKZF3*, *LRRC3C*, *MED24*, *ORMDL3*, *PSMD3*, *ZPBP2*
ENSG00000251301	*LINC02384*	25991924	*IL22*, *MDM1*
ENSG00000254211	*LINC01485*	25991924	*CPEB4*
ENSG00000256940	*AP001453.2*	25991924	*BAD*, *CCDC88B*, *DNAJC4*, *ESRRA*, *FERMT3*, *FKBP2*, *GPR137*, *KCNK4*, *MACROD1*, *NUDT22*, *PLCB3*, *PPP1R14B*, *PRDX5*, *STIP1*, *TEX40*, *TRMT112*, *TRPT1*, *VEGFB*
ENSG00000257069	*AP001453.3*	25991924	*BAD*, *CCDC88B*, *DNAJC4*, *ESRRA*, *FERMT3*, *FKBP2*, *GPR137*, *KCNK4*, *NUDT22*, *PLCB3*, *PPP1R14B*, *PRDX5*, *RPS6KA4*, *STIP1*, *TEX40*, *TRMT112*, *TRPT1*, *VEGFB*
Downregulated in UC			
ENSG00000227036	*LINC00511*	25991924	*SLC39A11*
ENSG00000170858	*LILRP2*	25991924	*KIR2DL1*, *KIR2DL3*, *KIR2DL4*, *KIR3DL3*, *LILRB1*, *LILRB4*
ENSG00000258867	*LINC01146*	25991924	*GALC*, *GPR65*, *KCNK10*
ENSG00000261266	*AC008870.3*	25991924	*CHP2*, *DCTN5*, *ERN2*, *NDUFAB1*, *PALB2*, *PLK1*
ENSG00000184809	*B3GALT5-AS1*	26937624	*B3GALT5*, *SH3BGR*
ENSG00000267130	*AC008738.2*	25991924	*CEBPA*, *CEBPG*, *LRP3*, *PEPD*, *SLC7A10*
Upregulated in CD			
ENSG00000241549	***GUSBP2***	27217703	
ENSG00000253838	*AC007991.2*	25991924	*ADAM2*, *IDO1*, *IDO2*
Downregulated in CD			
ENSG00000278217	***MALAT1***	27217703	*AP000769.1*, *EHBP1L1*, *FAM89B*, *FRMD8*, *KCNK7*, *LTBP3*, *MAP3K11*, *SCYL1*, *SIPA1*, *SSSCA1*
ENSG00000229005	*HNF4A-AS1*	29361088	*FITM2*, *GDAP1L1*, *HNF4A*, *R3HDML*, *TTPAL*
ENSG00000232693	*AC012370.1*	25991924	*SPRED2*
Upregulated in CD vs UC			
ENSG00000238164	*TNFRSF14-AS1*	25991924	*FAM213B*, *HES5*, *MMEL1*, *PANK4*, *PLCH2*, *TNFRSF14*, *TTC34*
ENSG00000269667	*AC092723.1*	25991924	*IRF8*

Forty-seven lncRNAs including 8 lncRNAs only from literature, 6 lncRNAs both from literature and our study, and 33 lncRNAs only based on our previous study were selected. Up- and downregulated lncRNAs in CD and UC, UC, CD and CD vs. UC (only upregulated) are categorized

Note: The non-IBD loci-associated lncRNAs are in bold

**Fig. 1:**
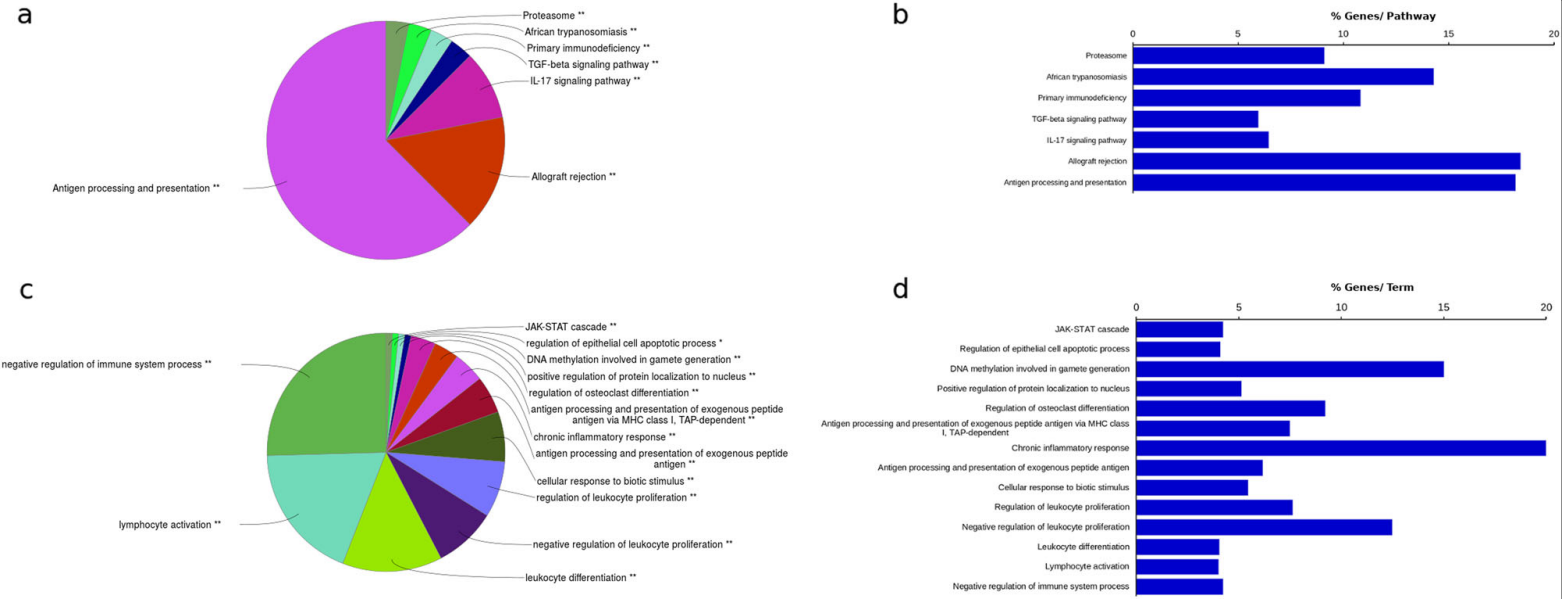
The guilt-by-association analysis of 47 IBD lncRNAs was performed by retrieving protein-coding genes (100 kb up/downstream) in the vicinity of these lncRNAs. One hundred ninety-three protein-coding genes were retrieved and used for gene ontology and pathway-based clustering using ClueGO in CytoScape. **a** shows the most significant biological process (BP) categories and **c** shows the most significant pathways based on KEGG annotations associated with the protein-coding genes. **b**, **d** Only the representative terms for each cluster as selected by ClueGo analysis based on the total number of genes and %Genes/Term are reported. The total number of genes and %Genes/Term are listed next to the representative term. The *P* values for each group are denoted by *P value < 0.05) and **P value < 0.01)

The guilt-by-association analysis of these 47 IBD lncRNAs was performed by retrieving protein-coding genes (100 kb up/downstream) in the vicinity of these differentially expressed lncRNAs. One hundred ninety-three protein-coding genes were retrieved and used for gene ontology and pathway-based clustering using ClueGO in CytoScape^78^. By performing functional annotation and gene ontology analysis, we identified an enrichment for various immune system processes in the GO analysis and an enrichment for primary immunodeficiency and various cytokine-mediated signaling pathways in the pathway analysis (Fig. 1). The detailed description of all enriched terms and pathways, including individual term and group *P* values, are provided in Supplementary Table 1. The enriched GO terms included negative regulation of immune system process, lymphocyte activation, leukocyte differentiation, the JAK-STAT cascade and antigen processing, and presentation via the major histocompatibility complex protein complex. The enriched pathways included TGF-β signaling, IL-17 signaling, allograft rejection and antigen processing, and presentation. Although the precise role of lncRNAs in IBD is poorly understood and must be further investigated, in line with our findings, compelling emerging evidence highlights the important role of ncRNAs in modulating immune responses and inflammatory cascades^32,33,79–83^.

The differential expression of lncRNAs in disease states is a valuable indication of phenotypic changes, which may have correlations with disease evolution. However, expression per se does not indicate the lncRNA’s functionality. Genomic conservation in other organisms suggests the importance of studied sequences for evolutionary pressure to preserve them, which may indicate functionality. Only 8 of the 47 IBD-associated lncRNAs evaluated here had orthologs in mouse and/or other organisms based on the HUGO comparison of orthology predictions (HCOP) database^84^ (Table 3). It is believed that due to the rapid evolvement of lncRNAs in species separated by >50 million years of evolutionary divergence, more than 70% of them have no sequence similar orthologs^85^. Although lncRNAs are poorly conserved in general, conservation of lncRNAs at the structural and functional levels might be substantially higher than their sequences. This is well established for other RNA molecules^86^. Our data indicate that while seven of eight lncRNAs have been conserved across mammalian species, GAS5 is the only one that is also conserved in zebrafish. However, poor genome annotations of many of the available organisms and technical errors in sample preparation may cause false negatives in searching for ortholog sequences in other organisms. Moreover, as noted, most of the conserved loci are in mice rather than other organisms that have a closer evolutionary relationship to humans. This may reflect better ncRNA annotation or more available data for the mouse genome than other genomes.

**Table 3:** List of lncRNAs that have orthologs in other organisms based on HCOP database

Name	Genome position (human)	Classification (human)	Orthologs (Ensembl/Entrez ID)
**GAS5**	Chr1:173,863,900–173,868,882	Processed transcript	• ENSMUSG00000053332; 14455 (mouse) • 81714 (rat) • 101883732 (zebrafish)
SMIM25	Chr20:50,267,486–50,279,795	lincRNA	• 100615910 (chimpanzee)
WFDC21P	Chr17:60,083,566–60,091,885	Processed transcript	• ENSMUSG00000051748; 66107 (mouse)
**DIO3OS**	Chr14:101,552,221–101,560,431	LincRNA	• ENSMUSG00000113581; 353504 (mouse) • 102546650 (rat)
IFNG-AS1	Chr12:67,989,445-68,234,686	Antisense	• ENSMUSG00000112230; 103214 (mouse)
H19	Chr11:1,995,163–2,001,470	LincRNA	• ENSMUSG00000000031; 14955 (mouse)
**UCA1**	Chr19:15,834,730–15,834,804	MiscRNA	• ENSMMUG00000045194 (macaque)
**MALAT1**	Chr11:65,502,914–65,503,008	MiscRNA	• ENSMUSG00000098462; ENSMUSG00000098869 (mouse) • ENSPTRG00000045406 (chimpanzee) • ENSMMUG00000047687 (macaque)

Note: The non-loci-associated lncRNAs are in bold

The guilt-by-association analysis confirms previous studies in which an overlap between the genetic loci associated with the risk for IBD and a large number of annotated lncRNAs was demonstrated^32,87^. In addition, it shows a pivotal role of lncRNAs in immune cells and system function regulation and the progression of IBD potentially through gene regulation. Consistent with these findings, it has been shown that IBD loci were significantly enriched and overlap active regulatory regions in primary intestinal epithelium and immune cells^88^. As IBD is an immune-mediated disease, these data not only reemphasize the importance of immune related processes in the disease pathogenesis and progression but also suggest the potential of significantly differentially expressed lncRNAs as biomarkers.

## LncRNAs as biomarkers for IBD

Currently, there is no single gold standard test available for the diagnosis of IBD. The routine diagnosis for IBD includes clinical symptom assessment combined with endoscopic examination, histology, serology, and radiology. Most of the current biomarkers reflect generalized inflammation and are not disease specific, which may lead to delayed treatment and further disease progression^89^.

LncRNAs have proven to be valuable diagnostic markers of various diseases^90,91^ because they are easy to obtain, stable, quick to detect by common molecular biology techniques (e.g., qRT-PCR), quantifiable, cost effective, and tissue/disease specific^90,92,93^. In IBD, in all discussed studies, the lncRNA profile data of colonic biopsy and blood samples show a clear difference between disease and healthy groups. Therefore, these findings should enable us to stratify not only the IBD subtypes but also the active and inactive states of the disease. However, additional studies are required to confirm these findings and identify new lncRNAs. Noninvasive body fluid-based lncRNA biomarkers provide an excellent window of opportunity to significantly improve our ability to diagnose and monitor IBD activity. While there are studies addressing the dysregulation of lncRNA profiles in the blood of IBD patients, it is evident that more data concerning dysregulated lncRNAs in other body fluids, including saliva, gastric juice, and urine, are needed.

Furthermore, the complexity of the pathological process of IBD and the limited knowledge available suggest that a single lncRNA biomarker may not be suitable for the diagnosis of IBD or its subtypes. Thus, a combination of several lncRNA candidates from different tissue sources, together with available biomarkers, may be necessary to provide a precise diagnosis. There are insufficient studies regarding the differentially expressed lncRNAs between early-stage and active IBD patients who can be used for early diagnosis. Overall, more studies with larger investigation/validation cohorts of IBD patients for both colonic biopsies and body fluids should be performed to increase the likelihood for introducing robust and reliable lncRNAs as biomarkers for IBD.

## Conclusion and future direction

Within the past few years, new insights into the functions of lncRNAs in many diseases have emerged. Many lncRNAs have shown promising translatable potential for clinical applications from prediction and diagnosis to treatment and monitoring of patients. However, in IBD settings, additional studies with larger cohorts and better techniques are required to have a higher likelihood of obtaining more significant outcomes. With a small population size, the obtained data are dispersed; therefore, the retrieved profile contains more lncRNAs, many of which may have little or no real association with the disease phenotype. In addition, the need for more advanced and robust techniques to detect lowly expressed lncRNAs and further studies to limit or address the impact of confounding factors, such as subject number, technical errors, and patient race, are as highly important as the previously outlined issues. At this stage, most of the studies in IBD merely show associations of lncRNAs with disease and/or disease subphenotypes and do not provide insight into the underlying mechanisms of lncRNAs in disease development.

To explore the role of lncRNAs in IBD pathogenesis and support their potential as biomarkers or therapeutic targets, further comparisons of body fluids and colonic tissue-specific lncRNAs, in addition to more thorough molecular and structure–activity relationship studies are necessary. There is only one study available that addressed this critical comparison for biomarker discovery. Although only three selected lncRNAs using only qPCR were investigated, a positive correlation between the detected lncRNAs from tissue and blood was detected^70^. lncRNAs hold substantial promise as therapeutic targets in different disease settings;^94,95^ however, there is an insufficient number of focused studies that address this issue in IBD. It is important to keep in mind that associations or correlations of lncRNAs with disease phenotypes do not provide information on the underlying molecular disease mechanisms per se. To unravel these mechanisms, more thorough mechanistic and functional studies that aim to investigate the biological roles are needed. However, undoubtedly in the near future, more research will investigate the therapeutic effect of targeting lncRNAs in IBD.

## Electronic supplementary material

Supplementary Table 1
